# The effect of rehabilitation interventions on physical function and immobility-related complications in severe stroke—protocol for a systematic review

**DOI:** 10.1186/s13643-018-0870-y

**Published:** 2018-11-17

**Authors:** Mark P. McGlinchey, Jimmy James, Christopher McKevitt, Abdel Douiri, Sarah McLachlan, Catherine M. Sackley

**Affiliations:** 1 School of Population Health and Environmental Sciences, Faculty of Life Sciences and Medicine, King’s College London, Addison House, Guy’s Campus, London, SE1 1UL England; 2 Physiotherapy Department, St Thomas’ Hospital, Guy’s and St Thomas’ NHS Foundation Trust, London, England

**Keywords:** Stroke, Stroke rehabilitation, Physical therapy speciality, Physical therapy modalities, Occupational therapy, Nursing, Recovery of function, Activities of daily living, Complications

## Abstract

**Background:**

Stroke rehabilitation aims to optimise function and reduce complications post-stroke. Rehabilitation to optimise physical function post-stroke has beneficial effects for survivors of mild to moderate stroke. However, little is known about the effectiveness of interventions to rehabilitate physical function or manage immobility-related complications for survivors of severe stroke. The systematic review aims to evaluate the effectiveness of rehabilitation interventions on physical function and immobility-related complications in severe stroke and identify topics for future research in this area.

**Methods:**

A systematic review of relevant electronic databases (MEDLINE, Embase, CINAHL, AMED, PEDro, DORIS and CENTRAL) between January 1987 and July 2017 will be undertaken to identify eligible published randomised controlled trials (RCTs) in any language. Ongoing RCTs will be identified by searching health-care trial registers (Stroke Trials Registry, ClinicalTrials.gov). Hand searches of identified study reference lists will also be performed. The PRISMA statement will be used to guide the systematic review. Two reviewers will screen search results, select studies using pre-defined selection criteria, extract data from and assess risk of bias for selected studies. Studies comparing the effect of one type of rehabilitation intervention to another or usual care on physical function and immobility-related complications for patients with severe stroke will be included. Studies may include participants with all levels of stroke severity but must provide sub-group analysis based on stroke severity. Studies will focus on any phase of the stroke rehabilitation pathway and will be grouped and analysed according to their timeframe post-stroke into acute and early sub-acute (up to 3 months post-stroke), early sub-acute to late sub-acute (from 3 to 6 months post-stroke) and chronic (greater than 6 months post-stroke). If sufficient studies demonstrate homogeneity, a meta-analysis will pool results of individual outcomes. The GRADE approach will be used to assess the evidence strength.

**Discussion:**

The results of this systematic review will summarise the strength of evidence for rehabilitation interventions used in the rehabilitation of physical function and immobility-related complications in severe stroke and identify gaps in evidence.

**Systematic review registration:**

The systematic review was registered with the International Prospective Register of Systematic Reviews (PROSPERO)—registration number CRD77737.

**Electronic supplementary material:**

The online version of this article (10.1186/s13643-018-0870-y) contains supplementary material, which is available to authorized users.

## Background

Stroke is the second most common cause of death and third most common cause of disability globally [1, 2]. In 2013, there were 10.3 million new cases of stroke, 6.5 million deaths and 113 million disability adjusted life years (DALYs) attributed to stroke worldwide [3]. The number of stroke survivors, estimated to be 25.7 million, is expected to rise over the next few decades primarily due to population growth and ageing [3]. In addition to the considerable number of people affected by stroke, stroke has a significant economic cost that includes the direct health-care costs to treat stroke, lost productivity and informal care costs [4, 5].

One method to manage the sequelae of stroke is stroke rehabilitation, a process that commences soon after stroke that enables a person to achieve their optimal physical, cognitive, communicative, emotional and social level of function [6–8]. Physical function can be defined as the ability to perform various physical activities and includes functioning of the upper limbs (e.g. grasping objects), lower limbs (e.g. walking), and activities of daily living (e.g. washing, dressing) [9, 10]. Rehabilitation of physical function comprises a large component of stroke rehabilitation programmes delivered by health-care professionals, such as physiotherapists and occupational therapists [11]. Whilst several systematic reviews support the use of rehabilitation interventions to improve aspects of physical function post-stroke, such as motor function, balance, walking speed and activities of daily living [12–14], it is not clear from these reviews if these interventions can be provided to stroke survivors with differing levels of stroke severity, particularly severe stroke.

Severe stroke can be understood as a stroke resulting in a significant amount of brain tissue damage and multiple neurological impairments, which leads to a significant loss of function [15]. Dependent upon how it is measured, it is estimated that between 14 and 31% of people who sustain a stroke globally have a severe stroke [16–20] and this cohort of the stroke population experience worse outcomes compared to survivors of less severe stroke [21–30]. In the initial hospitalisation phase post-stroke, they are more likely to develop acute medical complications, which are negatively associated with functional recovery [21]. In addition, as many as 40% of patients with severe stroke have died by 3 months compared to just under 5% for those patients with mild stroke [22–24]. Survivors of severe stroke spend longer in hospital, which results in increased hospital costs, and demonstrate slower and less functional recovery, which results in greater dependency upon hospital discharge [16, 17, 25–27]. For those who are discharged from hospital alive, severe stroke survivors are at least eight times more likely to be discharged to a nursing home [27, 28]. Longer-term care costs, which mostly support severe stroke survivors, represent 49% of total stroke care spending globally [5]. In the first-year post severe stroke, mortality can be as high as 60% [22] and survivors of severe stroke also experience very high levels of immobility-related complications, such as falls, contracture, pain, and pressure sores [29, 30]. Due to this residual disability, the physical assistance provided by caregivers to look after survivors of severe stroke as well as the psychosocial and emotional impact of the stroke on caregivers result in high levels of caregiver burden [31, 32]. Because of the range of issues faced by survivors of severe stroke, rehabilitation should focus on addressing these poor outcomes, particularly reduced physical function and its associated complications.

However, the extent to which rehabilitation can address these outcomes is not clear. As most studies investigating the efficacy of specific rehabilitation interventions on physical function included stroke survivors with mild to moderate levels of stroke severity [12–14], it is not known if research findings are applicable to survivors of severe stroke, who have very different clinical presentations compared to survivors of less severe stroke. It is not clear whether rehabilitation should focus more on the restoration of function, which may not always be possible or is often incomplete, or more on reducing immobility-related complications, which may reduce the longer term burden for caregivers of severe stroke survivors. Due to the lack of clarity in the literature regarding the optimal rehabilitation management for survivors of severe stroke, there is an urgent need to summarise evidence-based rehabilitation interventions designed to optimise physical function and reduce immobility-related complications for this cohort of the stroke population.

## Aims

This systematic review aims to:

- Establish the effectiveness of rehabilitation interventions on physical function and immobility-related complications for survivors of severe stroke.
- Identify questions for future rehabilitation research for survivors of severe stroke.

## Methods

The systematic review will be conducted in accordance with the Preferred Reporting Items for Systematic Reviews and Meta-analyses (PRISMA) statement [33]. The protocol was registered with the International Prospective Register of Systematic Reviews (PROSPERO) on September 22, 2017 (registration number CRD477737) [34]. The protocol has been reported according to the Preferred Reporting Items for Systematic Reviews and Meta-analysis Protocols (PRISMA-P) 2015 checklist (see Additional file 1).

### Eligibility criteria

Studies will be selected according to the eligibility criteria below and in Table 1, which are based upon the PICO (participant, intervention, comparator and outcome) format.

**Table 1:** Eligibility criteria for screened references

Study design	Randomised controlled trial (controlled trial)
Report characteristics	Full article Publication date January 1987 to July 2017
Participants	Adult humans and any of the following:
	• Severe stroke (measured using a recognised stroke-specific health measure, disability measure, functional measure) • Stroke with stroke severity subsets reported (at least 2 subsets with 1 subset identified as severe stroke)
Interventions	Any of the following:
	• Passive intervention (e.g. positioning, stretching programme, manual technique) • Active rehabilitation task (e.g. active or strengthening exercise, functional task practice) • Adjunctive therapies (e.g. electrical stimulation, treadmill training, robotics) • Aid or equipment (e.g. splint, wheelchair) • Environmental adaptation • Training and education (to carers or other health-care professionals)
	Provided by any of the following:
	• Physiotherapist, occupational therapist, nurse • Support or assistant staff of the above professions • Paid or unpaid carer
Comparator	Any of the following:
	• Any rehabilitation intervention • No intervention • Usual care
Outcomes	Any of the following:
	• Measure of body function, activity or participation • Immobility-related complication (any of the following): contracture, pressure sore, spasticity, chest infection, urinary tract infection, deep vein thrombosis, pulmonary embolism, fall, pain, shoulder pain, fatigue, depression Include outcomes measured by direct observation by health-care professionals as well as proxy reports by carers/patients

#### Participants

The review will include studies of adult stroke patients, defined as ≥18 years of age. As the review is investigating the effectiveness of rehabilitation interventions in severe stroke, studies may include patients with all levels of stroke severity but must include sub-group analysis based on stroke severity. Stroke severity will be defined using a score on either a validated and routinely used stroke specific health measure, e.g. National Institutes of Health Stroke Scale (NIHSS), Orpington Prognostic Scale (OPS); functional measure, e.g. Functional Independence Measure (FIM), Barthel Index (BI); or disability measure, e.g. Modified Rankin Scale (mRS) [35–39]. Whilst some measures, such as the OPS or mRS, use the same pre-defined cutoff scores to determine stroke severity [32, 35], other outcome measures, such as the NIHSS, BI or FIM, do not have as clearly defined cutoff scores to determine stroke severity. For the purposes of this review, an NIHSS score ≥ 16, BI score ≤ 45 (modified BI ≤ 9) and FIM score < 40 will be indicative of a severe stroke [16, 17, 40–44]. The mean/median severity scores and standard deviation/interquartile range of a study’s patient cohort must fall within the severe stroke category to be included in the study.

#### Interventions

The review will include studies that involve the provision of rehabilitation interventions used to manage problems relating to physical function or immobility-related complications post-stroke. For the purposes of the review, a rehabilitation intervention will be defined as any non-surgical or non-pharmacological intervention used in current clinical practice as part of the usual rehabilitative care of stroke patients. These interventions may be delivered by rehabilitation staff (e.g. physiotherapists, occupational therapists, nurses) or by people who have received training by rehabilitation staff, such as paid or unpaid carers of stroke survivors. It will not include interventions that primarily address problems related to cognitive, communicative or swallowing dysfunction post severe stroke. The list of interventions covered by the systematic review is in Table 1. The review will include studies of rehabilitation interventions that are provided in any phase of the post-stroke pathway and in any physical or geographical location. The review will note whether the authors have used a recognised framework to describe the intervention, such as the Template for Intervention Description and Replication (TIDier) checklist and guide [45].

#### Comparators

The review will include studies that have a comparator. The comparator will be any of the following: another type of rehabilitation intervention, usual care or no intervention. Usual care may be defined as the rehabilitation that the patient would normally receive as part of undergoing stroke rehabilitation.

#### Outcomes

The review will include studies that focus on the primary outcomes of physical function and post-stroke complications. As function can be defined according to body function, activities and participation [10], physical function will be assessed using measures of body function, e.g. Fugl-Meyer Assessment, Motricity Index, Modified Ashworth Scale; activity, e.g. BI, FIM, Motor Assessment Scale; and participation, e.g. Stroke Impact Scale [46–48]. An immobility-related complication may be defined as any medical problem arising after a stroke because of immobility or reduced physical activity [49]. These complications are listed in Table 1. The review will also note whether studies have included an economic evaluation.

### Study design

As the review will compare the effectiveness of one type of rehabilitation intervention to another, to usual care or no intervention, it will include studies where participants have been randomly allocated to one of two or more treatment groups. Therefore, it will include randomised controlled trials (RCTs) as the primary study design. If there are no RCTs that meet the eligibility criteria, quasi-experimental or non-randomised controlled trials will be considered for inclusion. However, the ability to establish the effectiveness of a physical rehabilitation intervention may be reduced with this type of study design and this will be specifically highlighted when reporting the results of the systematic review.

### Report characteristics

In order to avoid language or cultural bias, studies in any language or geographical location will be included. Studies not written in English will be translated into English using university-based translation services. Following a scoping review of the literature which demonstrated very few studies conducted before 2000 and to ensure studies reflect current clinical practice, the review will limit the search to 30 years (1987 to 2017).

### Information sources

Electronic searches of the following databases will be conducted: MEDLINE, Embase, Cumulative Index to Nursing and Allied Health Literature (CINAHL), Allied and Complementary Medicine Database (AMED), Physiotherapy Evidence Database (PEDro), Database of Research in Stroke (DORIS) and the Cochrane Central Register of Controlled Trials (CENTRAL). Databases will be searched from January 1987 to July 2017. Published abstracts from relevant stroke conferences (e.g. ESO, UKSF, SRR) will also be searched from January 1987 to July 2017. Ongoing studies will be identified by searching the Stroke Trials Registry (www.strokecenter.org/trials/) and ClinicalTrials.gov. These sources will be searched from 2012 to 2017 as it will be assumed that studies before these dates will have been completed and published. References from included studies will be hand searched and any potentially relevant study will be included for review. Forward citation checks of included studies will also be performed.

### Search

A search strategy has been developed that focuses on the following key search terms: severe stroke and stroke disability, stroke rehabilitation and physical rehabilitation (including individual interventions listed in Table 1), physiotherapy/physical therapy, occupational therapy, nursing care, functional recovery, physical function, activities of daily living and immobility-related complications (including individual complications listed in Table 1). Search terms have been established by scoping searches using Medical Subject Headings (MeSH). The MEDLINE search strategy is detailed in Appendix. This search will be adapted for the other databases.

### Data management

The results from the literature search will be uploaded to a reference management programme (Refworks). Duplicate references will be identified using the management programme and removed. A final list of non-duplicated references will be generated by one author (MM).

### Study selection

The titles and abstracts of the search results will be screened, and full text will be obtained for relevant studies. Two review authors (MM and JJ) will complete this process manually and independently, and any difference in opinion will be resolved by a third review author (CS). Full-text articles will be reviewed to determine if studies included through screening meet the inclusion criteria. An inclusion/exclusion checklist has been developed based on the eligibility criteria. Two review authors (MM and JJ) have piloted the checklist (Table 1).

### Data collection process and data items

Two review authors (MM and JJ) will independently perform data extraction for all eligible papers identified through the screening process. A data extraction proforma has been developed based on the Cochrane handbook for systematic reviews of interventions and the CONSORT statement for reporting randomised trials [50, 51]. The form focuses on study design and methods, participants, intervention details, outcome measures used, and results (Table 2). The form has been piloted independently by the lead author (MM). A Microsoft Excel document will be used to manage the data extraction.

**Table 2:** Data extraction form checklist

Source • Reviewer name • Review date • Study title and authors • Journal name • Publication date Eligibility • Confirm eligibility for review Introduction • Scientific background • Study aims and hypotheses included Methods—participants • Setting • Eligibility criteria • Stroke severity measure • Method of recruitment Methods—design and group allocation • Study design and duration • Group description • Sequence generation • Allocation concealment • Implementation • Blinding	Methods- Interventions • Description of intervention • Intervention details (timing, frequency, duration) • Resource requirements • Use of TIDier checklist—yes/no Methods—outcomes • Name and definition • Time points measured • Measure of function, activity or participation—use of validated outcome • Immobility-related complication—frequency of occurrence • Economic evaluation—yes/no Methods—statistical analyses used Results • Number of participants randomised/allocated per group/analysed • Details of any missing participants • Baseline demographics for each group • Summary data for each group at each time point • Compliance with intervention • Any adverse events Discussion/conclusion • Interpretation of results • Extent of generalisability • Key conclusions

### Risk of bias

Risk of bias of included studies will be performed by two review authors independently (MM and JJ). Information will be collected using the Cochrane Collaboration tool for assessing the risk of bias which focuses on sequence generation, allocation concealment, blinding, incomplete outcome data and selective outcome reporting [50]. An overall score will not be generated but a risk of bias judgement of ‘high’, ‘low’ or ‘unclear’ will be given for individual domains. Any difference in opinion will be resolved by a third review author (CS). The studies’ risk of bias will be presented in a table, and a narrative summary will be produced. Based on the assessment of risk of bias, a sensitivity analysis excluding studies with a high risk of bias will be performed.

### Data synthesis and analysis

If more than five adequately powered studies demonstrate homogeneity across studies in terms of rehabilitation interventions and outcomes, results for individual outcomes will be pooled quantitatively using both meta-analysis with fixed and random effect models and interpreted as appropriate. Possible sources of heterogeneity among studies, if observed, will be investigated. RevMan will be used as the meta-analysis software. Data from dichotomous outcomes, e.g. post-stroke complications will be analysed using risk ratio with 95% confidence interval. Data from standardised outcome measures of motor function and functional recovery will be treated as continuous data and will be analysed using mean differences with 95% confidence intervals. If studies demonstrate substantial differences in terms of data collected or only a few studies are found, a qualitative review of results will be performed. This will include a summary of study design, sample size, participant characteristics, outcomes and results. As there may be differences in recovery rates and outcomes according to the time post-stroke, a sub-group analysis will be conducted to explore the effects of time post-stroke. Studies will be grouped into three timeframes determined on the basis of when patients are recruited to the study: acute and early sub-acute (up to 3 months post-stroke), early sub-acute to late sub-acute (from 3 to 6 months post-stroke) and chronic (greater than 6 months post-stroke). These timeframes have been chosen based on recommendations for the standardised measurement of sensorimotor recovery in stroke trials [52].

### Quality of the evidence

Assessment of the strength of the evidence will be performed using the Grading of Recommendations Assessment, Development and Evaluation (GRADE) approach [50]. The five domains considered by the GRADE approach include risk of bias, inconsistencies between studies, indirectness, imprecision and publication bias. The quality of the evidence will be ranked high, medium, low or very low by two review authors independently (MM and JJ).

## Discussion

As survivors of severe stroke experience worse outcomes compared to survivors of less severe stroke, it is not clear if rehabilitation interventions designed to manage the residual physical problems for this cohort of the stroke population are effective. The results of this systematic review will summarise the strength of evidence for interventions used in the rehabilitation of physical function and immobility-related complications in severe stroke. If there is no, limited or inconclusive evidence for any of the identified interventions, the systematic review will identify where further research is required.

### Additional file

Additional file 1: Prisma-P checklist. (DOCX 33 kb)

## Appendix

### MEDLINE search strategy

1. exp Stroke/
2. severe stroke.mp.
3. stroke severit*.mp.
4. stroke disabilit*.mp.
5. exp Physical Therapy Modalities/
6. exp Occupational Therapy/
7. exp Nursing Care/
8. physical rehabilitation.mp.
9. exp Stroke Rehabilitation/
10. exp Patient Positioning/
11. exp Posture/
12. exp Exercise/
13. exp Exercise Therapy/
14. passive exercise.mp.
15. exp “Range of Motion, Articular”/
16. manual technique.mp.
17. active exercise.mp.
18. Resistance Training/
19. exp Muscle Stretching Exercises/
20. exp Electric Stimulation/
21. exp Electric Stimulation Therapy/
22. exp Wheelchairs/
23. seat?.mp.
24. exp “Equipment and Supplies”/
25. exp Teaching/
26. exp Education/
27. exp Motor Skills/
28. exp Movement/
29. motor function.mp.
30. motor recovery.mp.
31. exp “Recovery of Function”/
32. exp “Activities of Daily Living”/
33. functional independence.mp.
34. physical independence.mp.
35. complicatio*.mp.
36. exp Pain/
37. exp Contracture/
38. exp Pressure Ulcer/
39. exp Respiratory Tract Infections/
40. Muscle Spasticity/
41. Venous Thrombosis/
42. exp Pulmonary Embolism/
43. exp Urinary Tract Infections/
44. exp Accidental Falls/
45. exp Fatigue/
46. exp Depression/
47. 1 or 2 or 3 or 4
48. 5 or 6 or 7 or 8 or 9 or 10 or 11 or 12 or 13 or 14 or 15 or 16 or 17 or 18 or 19 or 20 or 21 or 22 or 23 or 24 or 25 or 26
49. 27 or 28 or 29 or 30
50. 31 or 32 or 33 or 34
51. 35 or 36 or 37 or 38 or 39 or 40 or 41 or 42 or 43 or 44 or 45 or 46
52. 47 and 48 and 49
53. 47 and 48 and 50
54. 47 and 48 and 51
55. limit 52 to (“all adult (19 plus years)” and randomized controlled trial)
56. limit 53 to (“all adult (19 plus years)” and randomized controlled trial)
57. limit 54 to (“all adult (19 plus years)” and randomized controlled trial)

## Abbreviations

AMED: Allied and Complementary Medicine Database
BI: Barthel Index
CENTRAL: Cochrane Central Register of Controlled Trials
CINAHL: Cumulative Index to Nursing and Allied Health Literature
DORIS: Database of Research in Stroke
FIM: Functional Independence Measure
GRADE: Grading of Recommendations Assessment, Development and Evaluation
mRS: Modified Rankin Scale
NIHSS: National Institutes of Health Stroke Scale
OPS: Orpington Prognostic Scale
PEDro: Physiotherapy Evidence Database
PRISMA: Preferred Reporting Items for Systematic Reviews and Meta-analyses
TIDier: Template for Intervention Description and Replication
